# Revalorization of Broccoli By-Products for Cosmetic Uses Using Supercritical Fluid Extraction

**DOI:** 10.3390/antiox9121195

**Published:** 2020-11-27

**Authors:** María Borja-Martínez, Jesús Lozano-Sánchez, Isabel Borrás-Linares, María A Pedreño, Ana B Sabater-Jara

**Affiliations:** 1 Department of Plant Biology, Faculty of Biology, University of Murcia, Campus de Espinardo, 30100 Murcia, Spain; maria.borja@um.es; 2 Department of Food Science and Nutrition, University of Granada, Campus of Cartuja, 18071 Granada, Spain; jesusls@ugr.es; 3 Research and Development of Functional Food Centre (CIDAF), Health Science Technological Park Avda. del Conocimiento nº 37, BioRegion Building, 18016 Granada, Spain; iborras@cidaf.es

**Keywords:** *Brassica oleracea* var. italica, antioxidant activity, by-products, bioactive compounds, cytotoxic activity, supercritical fluid extraction

## Abstract

The agri-food industry is currently one of the main engines of economic development worldwide. The region of Murcia is a reference area in Europe for the cultivation of fruits and vegetables and produces the bulk of Spanish exports of broccoli (*Brassica oleracea* var. italica). The processing of fresh produce generates a huge number of by-products that represent an important economic and environmental problem when discarded. In this work, an advanced extraction technique using environmentally friendly solvents was applied to assess the revalorization of broccoli by-products, by performing a comparative analysis with conventional extraction. To achieve this goal, supercritical fluid extraction based on response surface methodology was performed using CO_2_ and ethanol as solvents. The results obtained showed that the supercritical fluid extracts were rich in β-carotene, phenolic compounds, chlorophylls and phytosterols. Moreover, in bioactivity assays, the supercritical fluid extracts exhibited a high antioxidant activity and a cytoprotective effect in a non-tumorigenic keratinocyte cell line exposed to ultraviolet B light. The results indicate that supercritical fluid extracts from broccoli by-products could potentially serve as an ingredient for cosmetic purposes.

## 1. Introduction

Broccoli, a plant belonging to the *Brassicaceae* family, is cultivated in the region of Murcia, where the harvest represents 40% of the total broccoli production in Spain, with a total cultivated area of 12,500 ha and a production of 164,020 kg/ha [1]. Harvesting broccoli generates a huge amount of plant by-products, mainly leaves and stems, which constitute more than 95% of the harvested material [2].

In recent years, numerous studies have explored the use of by-products as a source of valuable bioactive compounds with potential application for the treatment and prevention of human diseases [3]. These bioactive compounds are derived from plant secondary metabolism and are synthesized in small amounts compared to other macronutrients. Different functions have been attributed to these valuable plant components. In plants they act as defense mechanisms against environmental stresses, and their biological properties may have health-promoting effects for consumers [4,5]. Among the main components of interest in broccoli by-products are glucosinolates and phenolic compounds (especially flavonoids and hydroxycinnamic acids), as well as carotenoids, sterols, vitamin C, fiber and mineral elements, which are essential for human health [6,7,8].

Nowadays, the market for natural cosmetics is growing, partly because of the high quality of the formulations, which attracts consumers, and because society is aware of the importance of sustainable development and protecting the environment. In this context, there is increasing interest in recovering bioactive compounds from vegetable by-products, not only to reduce the environmental impact of waste and the costs its treatment entails [9,10], but also to convert it into a particularly valuable source of extracts for cosmetic usage, due to the absence of residual pesticides and other potentially toxic chemical products [11]. In this regard, broccoli by-products can constitute a valuable source of health-promoting compounds with possible application in the cosmetic industry, mainly due to the antioxidant effect that these phytochemicals possess [12,13]. Therefore, the demand for high-value and ultrapure natural products is redirecting the efforts of the food industries towards the development of new green extraction methods that preserve the quality and quantity of bioactive compounds without a negative environmental impact [14,15].

A new attractive alternative method for extracting bioactive compounds involves the use of supercritical fluids. It has been demonstrated that supercritical fluid extraction (SFE) overcomes many of the limitations of conventional extraction (CE) techniques, avoiding the use of potentially toxic organic solvents, and can be applied to obtain functional and nutraceutical components from different natural sources [16]. Other advantages include shorter extraction times, increased yields, the use of safe, environmentally friendly solvents and little or no solvent residue, which render the method suitable for heat-labile food processing. Additionally, the method does not induce oxidation or degradation, the costs of solvents and their storage are reduced and specific bioactive compounds can be obtained by regulating the solvent, temperature and pressure [17]. Thus, this extraction technology is considered particularly useful as it allows bioactive compounds with different characteristics to be obtained separately from the same plant biomass.

In this work, a supercritical fluid extraction procedure was optimized for the recovering of bioactive compounds from Parthenon and Naxos broccoli by-products (leaves and stems) (*B. oleracea* var. italica). To achieve this goal, a response surface methodology (RSM) was developed to optimize the SFE procedure. Extracts obtained using these methodologies were characterized and their bioactivity was verified using an antioxidant assay and a non-tumorigenic keratinocyte cell line.

## 2. Materials and Methods

### 2.1. Chemicals and Reagents

Folin–Ciocalteu’s reagent, gallic acid, β-carotene, phytosterols, chlorophyll a and b, α-tocopherol, quercetin, Trolox (6-hydroxy-2,5,7,8-tetramethyl-2-carboxylic acid), ABTS (2,2′-Azino-bis (3-ethylbenzothiazoline-6-sulfonic acid) diammonium salt), and MTT (3-(4,5-dimethylthiazol-2yl)-2,5-diphenyl-2H-tetrazolium bromide) were purchased from Sigma-Aldrich (Taufkirchen, Germany). Ethanol and dimethyl sulfoxide (DMSO) were of analytical grade. Dulbecco’s Modified Eagle’s Medium (DMEM) was from Invitrogen (Carlsbad, CA, USA). CO_2_ and ethanol, used as solvents for the SFE, were provided by Air Product and Chemicals (Allentown, PA, USA) and VWR chemicals (Radnor, PA, USA), respectively. Ottawa sand was provided by Fisher Scientific (Leicester, UK) and glass wool, also used in extraction procedures, was acquired from Sigma–Aldrich (Taufkirchen, Germany).

### 2.2. Sample Preparation

Broccoli by-products (leaves and stems) from the cultivars Parthenon and Naxos (*B. oleracea* var. italica) were provided by Agrícola Santa Eulalia S.L (Murcia, Spain). Leaves and stems were homogenized and mixed according to the ratio 1:1, 3:1 and 1:3 (leaf:stem). Prior to dehydration, the leaves, stems and their mixtures were cut to a size of 10 × 10 mm using a Urschell^®^ Model G-A Dicer and then ground into a uniform powder using a Comitrol^®^ Processor Model 1700 Urschell. Once the by-products were cut and crushed, they were dried in a climatic chamber at 55 °C for 24 h until reaching a final moisture content of 4%. Finally, samples were ground to achieve a uniform powder. For further analysis, the ground samples were stored at room temperature and in darkness until the analysis.

### 2.3. Conventional Extraction

Bioactive compound extractions from dried broccoli by-products were performed using different sources of plant material, i.e., leaves, stems and separately or in combination. In order to compare the bioactive compounds present in the SFE extracts with those obtained from conventional extracts, the CE of carotenoids, chlorophylls, total phenolic compounds and phytosterols was carried out using the methodology described by Miras-Moreno et al. [18]. Briefly, dried broccoli by-products were incubated with methanol:Tris-HCl (50 mM, pH 7.5) (1:1; *v*/*v*) in an ice bath and in darkness for 20 min at 4 °C. Then, chloroform (1:1, *v*/*v*) was added and the samples were centrifugated at 15.000× *g* for 5 min. The organic phase was collected and evaporated at 40 °C in a rotavapor (Rotavapor^®^100, Buchi). The dried extracts were then dissolved in ethanol and filtered through 0.2 µm nylon syringe filters and stored at −20 °C until further analysis. The extraction yield (EY) was calculated as the percentage of total mass of dry extracts per gram of raw material extracted used over the extraction procedures (EY = (amount of dry extract/amount of raw material) × 100).

### 2.4. Supercritical Fluid Extraction

SFE was carried out with a Waters Prep Supercritical Fluid Extraction system (SFE-100Waters^®^, TharSFC, Thar Technologies, Inc., Pittsburgh, PA, USA) equipped with CO_2_ and co-solvent P-50 pumps, two pressure heating exchangers (low and high), a pressurized extraction vessel (100 mL), two pressurized collection vessels, and three automated back pressure regulators. The SFE system was connected to an Accel 500 LC chiller by Thermo Scientific (TharSFC, Thar Technologies, Inc., Pittsburgh, PA, USA).

Broccoli by-product powder was loaded into the extraction vessel as follows: 15 g of sample (leaves/stems, 3:1) was mixed in a ratio of 2:1 with sea sand, and glass wool was added at the top and bottom of the extraction vessel to prevent sample loss. Each procedure was accomplished in a dynamic mode. All extracts obtained were evaporated at 35 °C in a rotavapor (Rotavapor^®^100, BÜCHI Labortechnik AG, Flawil, Switzerland)) and stored at −20 °C until analysis. The dried extracts were reconstituted in ethanol at a concentration of 1000 µg extract mL^−1^, filtered through 0.2 µm nylon syringe filters and stored at −20 °C until further analysis. EY was calculated as described in Section 2.3.

### 2.5. Quantification of Bioactive Compounds in SFE and CE Extracts

#### 2.5.1. Chlorophylls and Carotenoids

SFE and CE extracts were dissolved in ethanol and the absorbance at 450, 648.6 and 664.2 nm was measured in a UV-visible spectrophotometer (JASCO V-730, Easton, MD, USA). A β-carotene calibration curve was prepared in ethanol with concentrations ranging from 1 to 80 μg mL^1^ and the absorbance was measured at 450 nm. The β-carotene total content in the samples was calculated from the linear regression of the calibration curve (*R^2^* = 0.9992). Likewise, the carotenoids were identified by comparison with commercial standards using a Jasco LC-NetII/ADC system with a Zorbax Eclipse XDB-C18 end capped 5 µm, 4.6 × 150 mm reverse phase column (Agilent Technologies, Palo Alto, CA, USA) as described by Fatimah et al. [19]. Chlorophylls a and b were determined using the equations previously described by Sumanta et al. [20] from the absorbances at 648.6 and 664.2 nm, which allowed the results to be expressed as mg per g of dried extract.

#### 2.5.2. Total Phenolic Content

The total phenolic content (TPC) was determined using the Folin–Ciocalteu method [21] with modifications. In brief, 10 μL of extracts, resuspended in an adequate volume of ethanol, were mixed with 790 μL of distilled water and 50 μL of undiluted Folin–Ciocalteu reagent. After 3 min of reaction, 150 μL of sodium carbonate 20% (*w*/*v*) was added and the mixture was incubated in darkness at room temperature for 2 h. After this time, the absorbance was measured at 760 nm using an UV-VIS spectrophotometer (JASCO V-730, Easton, MD, USA). A calibration curve of gallic acid standard solutions ranging from 0.01 to 10 μg mL^−1^ (*R^2^* = 0.9993) was used to quantify the TPC, which is expressed as mg of gallic acid equivalents (GAE) per g of dried extract.

#### 2.5.3. Phytosterols and α-Tocopherol

Identification and quantification of phytosterols and α-tocopherol was based on mass spectra obtained by gas chromatography (GC), using an Agilent Technologies 6890 Network GS System, equipped with a mass selective detector (Agilent Technologies 5973) as described by Almagro et al. [22]. A capillary column 30 m × 0.25 mm (Agilent 19091 S-433HP-5MS) was used for GC/mass spectrometry (MS) analysis. The oven temperature was initially set at 60 °C and increased up to 310 °C at a rate of 10 °C min^−1^. A constant flow rate of 0.1 mL min^−1^ using helium as the carrier gas was used. The mass range was monitored from m/z 50–800, with an ionization energy of 70 eV. The injection volume was 1.0 µL. The identification of different phytosterols (campesterol and β-sitosterol) and α-tocopherol was confirmed by comparing (match > 90%) the obtained experimental MS spectra with those provided by the NIST spectral library database. The concentration was estimated based on an adequate standard curve using the respective standard. Data were acquired and processed with Chemstation software.

### 2.6. Antioxidant Capacity

The antioxidant capacity of the extracts obtained from the broccoli by-products was determined using the Trolox Equivalent Antioxidant Capacity (TEAC) method or ABTS method. The anti-radical capacity was evaluated by analyzing the effect of CE and SFE extracts on the stable free radical ABTS^.^ +. The activity of the samples on ABTS + was monitored spectrophotometrically at 414 nm according to the protocol described by Escribano et al. [23]. The ABTS + radical was prepared from a 20 mM stock of ABTS using peroxidase (1 mg mL^−1^ commercial horseradish peroxidase type VI, obtained from Sigma) in the presence of hydrogen peroxide and 0.2 M sodium acetate buffer, pH 5.0. Reactions were carried out in 0.2 M sodium phosphate buffer, pH 7.0. Measurements were made at 414 nm in 1.5 mL plastic cuvettes at time 0 and after 24 h of incubation at 20 °C in the dark, on a Jasco V-730 spectrophotometer. All experiments were run in triplicate. TEAC is expressed as mg of Trolox equivalent per g of extract.

### 2.7. Cytotoxic Evaluation

#### 2.7.1. Cell Cultures and Treatments

The human immortalized non-tumorigenic keratinocyte cell line (HaCaT) was purchased from CLS Cell Lines Service (Eppelheim, Baden-Württemberg, Germany) and maintained in DMEM supplemented with 4.5 g L^−1^ glucose, 2 mM L-glutamine and 10% fetal bovine serum. Cells were cultured in a humidified incubator containing 5% CO_2_ at 37 °C. HaCaT cells were seeded at a density of 5 × 10^3^ cells per well in 96-well microplates and incubated for 24 h to reach 80% confluency. All the experiments were carried out after this time.

As the extracts obtained from the SFE are organic solvent-soluble, the extracts obtained by optimized SFE from Naxos broccoli by-products (SFE-NX) were dissolved in two solvents: ethanol and dimethyl sulfoxide (DMSO). First, the toxicity of both solvents was tested. For this, HaCaT cells were seeded in a 96-well microplate and left overnight to allow cell attachment. After 24 h, the solvents were added to the wells at different concentrations (ranging from 0 to 1%) in the culture media. After 24 h, the cell viability was determined using the MTT method, which expresses the percentage of live cells compared to the control.

To evaluate the cytotoxic activity of optimized SFE-NX extracts, HaCaT cells were seeded at the same cell density as described above. After 24 h, optimized SFE-NX extracts ranging from 0.1 to 200 μg mL^−1^ in DMSO were added to the cells for 12, 24 and 48 h. After that, the cell viability assay was performed.

Finally, the protective effect of the SFE-NX extracts against ultraviolet light B (UVB) was evaluated. First, the effect of UVB light on the cell viability was assayed. After that, HaCaT cells were maintained for 12 h in the presence of different concentrations of SFE-NX extracts (0, 0.1, 1 and 10 µg mL^−1^). Quercetin (30 µg mL^−1^) was used as a positive control. The culture media containing the extracts was then discarded and replaced by a thin layer of phosphate buffer solution before the UVB light exposure. HaCaT cells were exposed to three specific doses of UVB light, 0, 25 and 50 mJ cm^−2^, using a UVB lamp inside a laminar flow cabinet (Vilber Louvert Biolink™ BLX UV Crosslinker,, Eberhardzell, Germany). The treatment time varied to provide the specific doses of UVB light. After irradiation, PBS was withdrawn and replaced by growth medium, and cell viability was determined after 24 h.

#### 2.7.2. Cell Viability Assay

The cytotoxicity of the optimized SFE-NX extract was determined in HaCaT cells using the colorimetric MTT assay.

After cell exposure to SFE-NX extracts and/or UVB light, the culture medium was removed and replaced with 200 μL of a filtered 1 mg mL^−1^ MTT solution and the cells were then incubated in darkness at 37 °C/5% CO_2_ for 4 h. The MTT solution was withdrawn from the wells and replaced with 100 μL of DMSO to dissolve the violet formazan crystals. After 5 min of gentle shaking, the absorbance was measured at 570 and 690 nm using a microplate reader FLUOstar Omega (BMG Labtech, Ortenberg, Germany). Cell viability was calculated as the percentage of viable cells compared to the control. The 50% growth inhibitory concentration (IC_50_) was calculated from the time course obtained by plotting the percentage of cell viability (compared to the control) versus different concentrations of SFE-NX extracts.

### 2.8. Statistical Analysis

In addition to the statistical analysis performed to optimize SFE, data from bioactive compounds and cytotoxic activity were analyzed by one- or two-way analysis of variance (ANOVA), respectively, followed by a Tukey’s test in order to ascertain the significance of the observed differences using the SPSS package (SPSS Inc., Chicago, IL, USA) version 22.0, and *p* values < 0.05 were considered as statistically significant.

## 3. Results and Discussion

### 3.1. Screening for Bioactive Compounds in Broccoli by-Products

**Figure 1:**
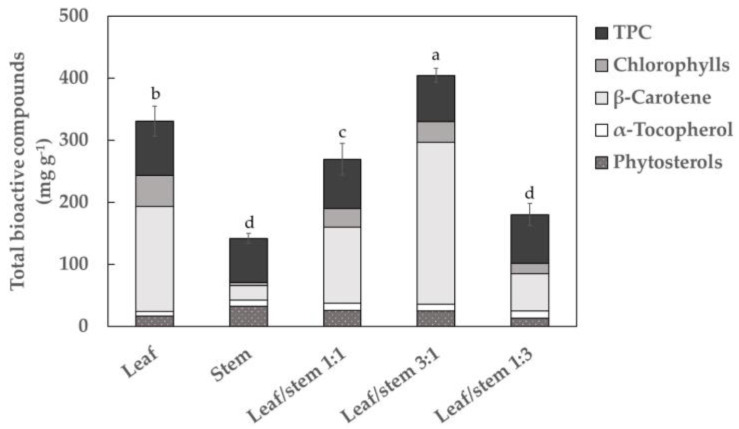
Total bioactive compound contents (mg per gram of extract) in the Parthenon broccoli cultivar by-products (leaves, stems and leaf/stem) in conventional extracts. Values are given as the mean ± SD of three replicates. Different letters (a–d) denote statistically significant differences according to Tukey’s test (*p* < 0.05). TPC: total phenolic compounds.

Methodologies to extract bioactive compounds from plant by-products have been developed to increase the extraction yield, efficiency and selectivity. Prior to SFE optimization, the bioactive compounds in Parthenon broccoli by-products were determined by applying CE to different mixtures of plant material: leaves, stems and a mixture of leaves and stems in different proportion (Figure 1). The total content of bioactive compounds obtained after performing the extraction from the different parts of the plant is shown in Figure 1. The highest total content was observed in the mixture of leaf/stem 3:1 (392.22 ± 10.51 mg g^−1^ extract) which is representative of the total volume of by-products generated in broccoli crop, followed by leaves (330.76 ± 24.41 mg g^−1^ extract). The lowest total bioactive compounds were observed in stems (134.75 ± 8.46 mg g^−1^ extract); the respective extraction yields (EY) were 2.57, 2.52 and 1.13%. The predominant compounds were β-carotene (~65%) and TPC (~19%). Minor bioactive compounds were chlorophylls, phytosterols and α-tocopherol, which represented less than 8%. Based on the results and to optimize the utilization of broccoli by-products, a leaf-stem mixture (3:1) was selected as the raw material for further studies, with the aim to obtain the highest recovery of phytochemicals.

The extraction of target compounds depends on different factors such as solvent composition, extraction time and ratio of raw materials used to carry out the procedure [24,25,26]. Since the solvent composition and extraction time were keeping constants in all the experiments, these results could be justified by the raw material composition. Indeed, the efficiency of CE methods can also be affected by the permeability of the tissues to the solvents used to recover bioactive compounds [27,28]. Leaves and stems were homogenized and mixed according to the ratios 1:1, 3:1 and 1:3 (leaf/stem). After that, they were crushed and dehydrated using the same experimental conditions (see Materials and Methods section). Results pointed out that the minor total bioactive concentration was obtained in stems. According to the scientific literature, in highly lignified tissues such as stems, the accessibility of solvents to the target compounds is reduced, increasing the resistance to extraction of phytochemicals and therefore reducing its recovery [28]. This fact could explain the low content of bioactive compounds found in the stem extracts. Consequently, an increase of stem in the broccoli by-products mixture generated a decrease in the total bioactive compounds recovery, as it has been described in Figure 1 (ratio 1:1 and 1:3, leaf:stem). However, the best results were obtained using the leaf/stem proportion of 3:1 (m/m) in comparison with both pure raw materials. This behavior could be explained by the fact that the combination of stem in lower concentration with leaves in the sample mixture could improve the isolation of the phytochemical fraction since stem tissues could act as a dispersant, thus allowing better accessibility of solvent to the leaf tissues.

### 3.2. Optimization of the SFE Conditions

To establish the optimal conditions described in Materials and Methods, an experimental design based on RSM was developed. RSM allows the behavior of the response variable under the evaluated experimental conditions to be summarized. Considering that β-carotene was the most representative bioactive compound in the broccoli by-products, it was selected as the target analyte to optimize SFE.

Independent variables were selected according to the factors and principles of the procedure that govern bioactive compound recovery: temperature, pressure, co-solvent percentage, flow rate and time. The effect of SFE parameters on β-carotene recovery from broccoli by-products was evaluated according to a central composite design (CCD) 2^5^ with two central points and three levels (−1, 0, 1) for each independent variable: (a) temperature, 40, 60 and 80 °C; (b) pressure, 150, 300 and 450 bars; (c) co-solvent percentage, 7, 18.5 and 32%; (d) flow rate, 28, 30 and 32 g min^−1^; and (e) time, 40, 60 and 80 min. A total of 26 experiments were carried out in a randomized order (Table 1). To ensure the reproducibility of each of the conditions tested, the experiments were carried out in duplicate.

Statgraphics Centurion software XVI provided by Statpoint Technologies (Warrenton, VA, USA) was used to process the obtained results. ANOVA tests were performed for each proposed factor to evaluate the significant (*p* < 0.05) or non-significant (*p* > 0.05) effect [29]. Table 2 includes fitting parameters for the proposed CCD 2^5^ model and the statistical significance of the independent variable effects. To facilitate the interpretation of the model, only the individual effects and the quadratic and interaction effects between variables with a significant impact are included in the table.

**Figure 2:**
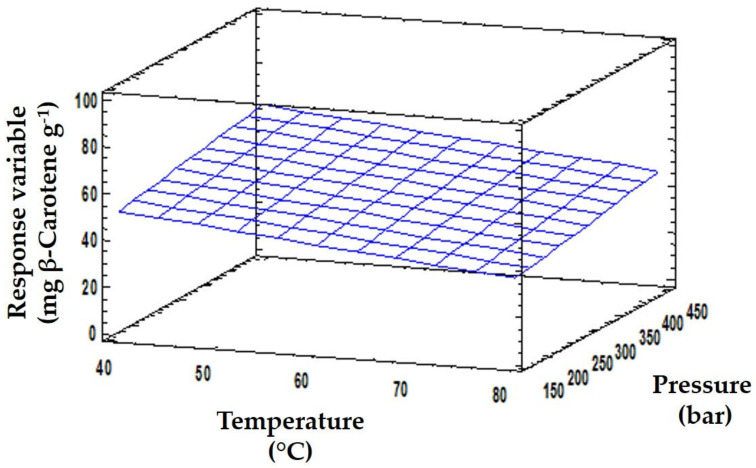
The 3D predicted response surface plots of the effects of temperature and pressure on the β-carotene content.

Regarding the parameter fitting, the model presented a high degree of correlation (*R^2^* = 0.935). The result explains the variance within the data, as this value evaluates the ability of the model to predict the behavior of the response variable. Furthermore, the lack of fit was not significant (*p* > 0.1). This statistical parameter verifies the fitting quality of the model. Therefore, this model provided a good approximation to the experimental conditions (Figure 2), which was confirmed by the parameters obtained by ANOVA analysis (Table 2).

Once the model fitting was verified, the optimization enabled the maximization of the evaluated response. For this purpose, *p*-values of linear, quadratic and interaction effects among independent variables were analyzed. Temperature, flow rate and extraction time had significant effects, showing a *p*-value of 0.0452, 0.0341 and 0.0402, respectively (*p*-value ≤ 0.05). With respect to pressure, the parameters were also not significant at the level of 90% (*p*-value = 0.0664). These statistically significant results proved that these variables had an individual effect on the β-carotene content. Although the ethanol percentage did not significantly affect the value of the response variable, statistical treatment indicated that the interaction between ethanol and time had a significant impact on the β-carotene content. The quadratic effect of the flow rate was also significant (*p*-value = 0.0479).

After analyzing the CCD 2^5^ model, a quadratic polynomial model (Equation (1)) was used to fit the experimental data:

(1) $$Y=\;\alpha 0\;+\;\overset{k}{\underset{\;i\;=\;1}{\sum }}\alpha_{i}X_{i}+\;\overset{k}{\underset{i=1\;}{\sum }}\alpha_{ii}\;X_{i}^{2}+\;\overset{k}{\underset{i=1}{\sum }}\overset{k}{\underset{j=i+1}{\sum }}\alpha_{ij}\;X_{i}\;X_{j}$$

*Y* = β-carotene content; *α*_0_, constant coefficient, which fixed the response at the central point of the design; *α_i_*¸ *α_ii_* and *α_ij_* regression coefficients of the linear, quadratic and interaction effects, respectively; *X_i_* and *X_j_* value of independent variables. After fitting the experimental data to a reduced model and keeping only the significant parameters in the quadratic model, the resulting model equation was as follows (Equation (2)):

(2) $$Y=-2478.19-0.407818\;+\;0.0376639\;+\;2.55145\;+\;159.064\;+\;1.20856X_{5}-0.0408957X_{3}X_{5}-2.56244X_{4}X_{4}$$

Using the equation to explain the model, an optimization of SFE conditions was proposed to maximize β-carotene recovery. The optimum values predicted by the model were: 40 °C, 443 bar, 7% ethanol, 31 g min^−1^ of flow rate and 68 min. The proposed optimal conditions were applied to confirm the theoretical results. Under these extraction conditions, the β-carotene content of the SFE extract was 84.11 ± 1.51 mg β-carotene g^−1^ dried extract. Regarding the selection of temperature, Baysal et al. [30] carried out an optimization study of this parameter in tomato paste waste to obtain carotenoids. Their results showed that treatment with temperatures above 65 °C gave the highest EY, although due to the risk of carotenoid degradation, a lower temperature was advocated. To reduce the thermal degradation of these compounds, Alzate et al. [31] suggested using temperatures below 40 °C, in agreement with our results (Figure 2). In relation to pressure, the optimized range for broccoli by-products is similar to that described by Vega et al. [32] for the extraction of carotenoids from carrots. These authors reported that the use of extraction pressures of around 400 bars increased the yield in carotene extraction, using a temperature of 40 ºC and 5% of ethanol as a cosolvent. Therefore, the results we obtained with SFE based on RSM are within the optimal conditions described by other authors for the extraction of β-carotene.

In addition, the predictable value for β-carotene was calculated based on the obtained equation (Equation (2)) and compared with the experimental value obtained when the design and optimal conditions were applied (Table S1). Analysis of the results revealed that, with the exception of SFE10, the variance of the data was acceptable, being in the range of 0.4 to 20 (CV ≤ 20%). Indeed, the variance between theoretical and optimized experimental data was very slight (CV = 0.4%), indicating a good reproducibility of the investigated systems [29].

### 3.3. Quantification of Bioactive Compounds from Optimized SFE Conditions

**Figure 3:**
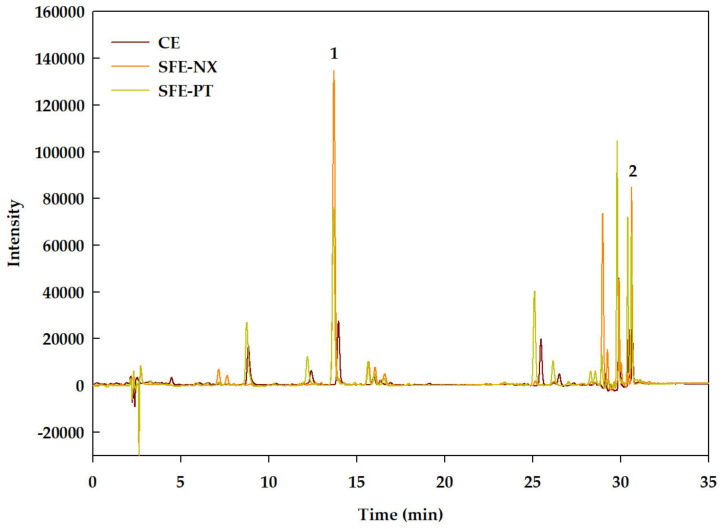
HPLC-DAD profile of the optimized supercritical fluid (SFE) and conventional (CE) extracts derived from Parthenon (SFE-PT) and Naxos (SFE-NX) broccoli by-products, acquired at 454 nm. 1: Lutein; 2: β-Carotene.

**Figure 4:**
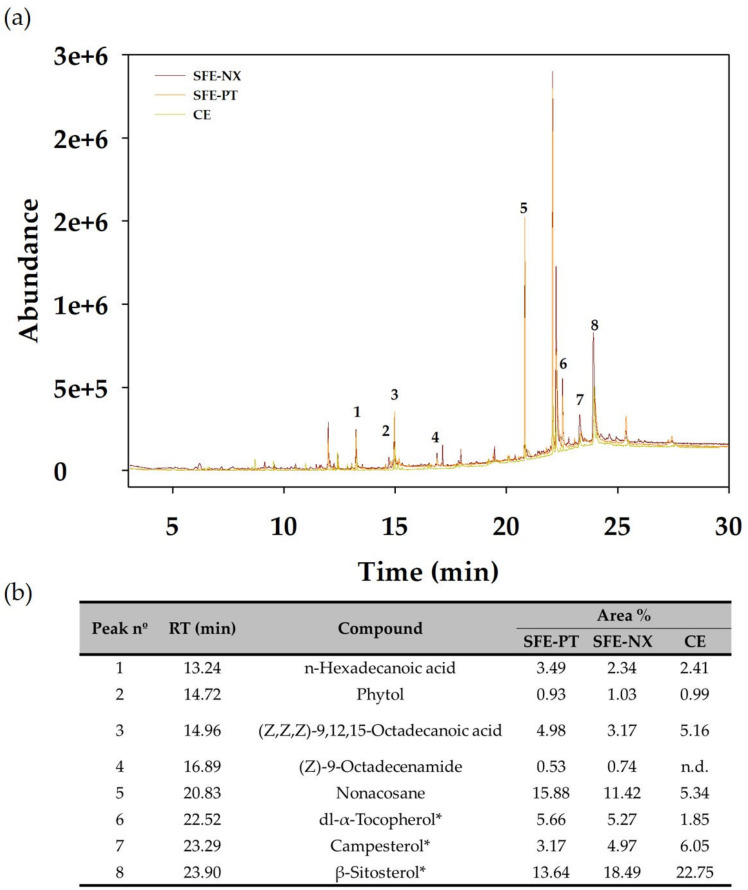
(**a**) Total ion chromatogram (TIC) of the optimized supercritical fluid (SFE) and conventional (CE) extracts derived from Parthenon (SFE-PT) and Naxos (SFE-NX) by-products. (**b**) Identified compounds in the extracts obtained from broccoli by-products. * denote the quantified compounds; RT: retention time; n.d.: not detected.

The optimized SFE conditions were also applied to the extraction of bioactive compounds from the Naxos broccoli by-products and the bioactive compound contents in the optimized extracts were analyzed by HPLC-DAD (Figure 3) and GC-MS (Figure 4). The HPLC analysis (Figure 3) revealed the presence of the xanthophyll lutein and β-carotene, which have been previously identified as the main carotenoids in *Brassica* species [33,34]. Predominant among the compounds identified by GC-MS (Figure 4) were the phytosterols campesterol and β-sitosterol, and α-tocopherol. Phytol and fatty acids were also detected.

Table 3 shows the major bioactive compounds, total bioactive compounds and EY found in the CE extracts and in the optimized SFE extracts from the by-products of cultivars Parthenon (SFE-PT) and Naxos (SFE-NX). Total bioactive compounds in SFE-NX extracts were 1.47-fold higher compared to SFE-PT (Table 3). However, the proportion of the different compounds varied depending on the cultivar. Thus, while the proportion of β-carotene was higher in SFE-NX extracts (62.74% versus 52.03% in SFE-PT extracts; Table 3), the proportion of phytosterols and chlorophylls was 2.28- and 1.17-fold higher, respectively, in SFE-PT extracts and no differences were observed in TPC between the two cultivars (around 12%) (Table 3).

At this point, it should be noted that the total bioactive compounds obtained by CE methods (Figure 1) from Parthenon by-products was significantly higher (392.22 ± 10.51 mg g^−1^ extract) than obtained by the SFE method (161.22 ± 6.35 mg g^−1^ extract) (Table 3). However, the EY obtained by SFE was comparable to that of the CE method (2.61 and 2.57%, respectively) (Table 3). Moreover, the drawbacks of CE, such as the use of non-environmentally friendly solvents, proliferation of steps (maceration, centrifugation and evaporation) as well as an incomplete removal of solvents, longer extraction times and a high solvent consumption, make the SFE process a good option to obtain bioactive compounds from broccoli by-products. The application of SFE involves only one step, uses green solvents, and part of the solvent mixture is removed in the depressurization step. Arnáiz et al. [8,35] demonstrated that broccoli by-product extracts obtained by SFE contained a higher percentage of unsaturated fatty acids (especially the polyunsaturated 18:3) as well as amino acids (proline and glutamine), when compared with Soxhlet and conventional solvent extraction, respectively.

### 3.4. Antioxidant Activity of Extracts Obtained by SFE

Once the bioactive compounds in the optimized SFE extracts from Parthenon and Naxos by-products were quantified, the antioxidant activity of extracts (expressed in terms of Trolox equivalents (mg Trolox g^−1^)) was measured by the TEAC method. The highest value of antioxidant activity was obtained for the Naxos cultivar (338.69 ± 31.95 mg Trolox g^−1^ versus 262.57 ± 40.57 mg Trolox g^−1^ in the Parthenon cultivar), which could be correlated with the higher content of total bioactive compounds in the optimized SFE-NX extracts (Table 3). Furthermore, the high antioxidant activity of these extracts could be due to a synergistic effect of the total bioactive compounds. These results support those of Arnáiz et al. [36], who found that SFE extracts obtained from Naxos leaves provided the highest antioxidant activity (using the TEAC method) and TPC, while the lowest value was obtained from Parthenon leaf extracts. Likewise, Hwang and Lim [9], using broccoli by-products (leaves and stems) from different cultivars and maturity stages at harvest, demonstrated that these waste products contained high levels of phenolics, which was correlated with a high antioxidant and anticancer activity.

### 3.5. Cytotoxic Activity

**Figure 5:**
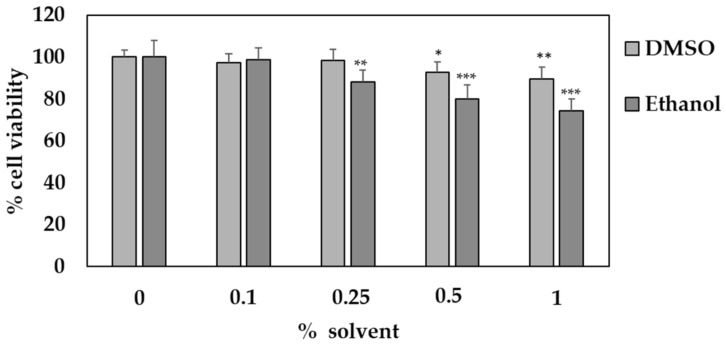
Effect of different percentages of dimethyl sulfoxide (DMSO) and ethanol on HaCaT cell viability. Cell viability was determined using an MTT assay and is presented as a percentage of the control. Values are given as the mean ± SD of three replicates. * *p* < 0.05; ** *p* < 0.01, *** *p* < 0.001.

The cytotoxic effect of the optimized SFE-NX extract on HaCaT human skin cells was evaluated using the MTT method, as this extract contained the highest bioactive compound content (231.73 ± 17.23 mg g^−1^, Table 3) and antioxidant activity (338.69 ± 31.95 mg Trolox g^−1^). Beforehand, the HaCaT cells were treated for 24 h with different concentrations of ethanol and DMSO to ascertain the minimum amount of each organic solvent that did not cause a significant reduction in cell viability (Figure 5). HaCaT cells showed a significant decrease in viability to 87% and 92% when exposed to 0.25% of ethanol and 0.5% DMSO, respectively. Consequently, and because it showed a higher solubility for the extracts, DMSO was selected for the subsequent experiments and used at a concentration of less than 0.5%.

**Figure 6:**
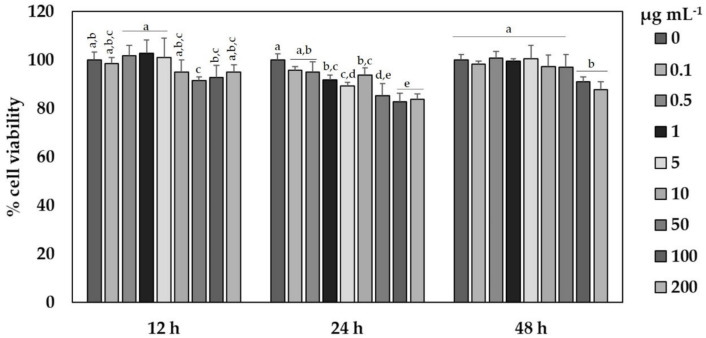
Time course of cytotoxic effect on the viability of HaCaT keratinocytes of different concentrations (0–200 µg extract mL^−1^) of the optimized SFE extracts derived from Naxos (SFE-NX) by-products. Cell viability was determined using an MTT assay and expressed as percentages of the control. Values are given as the mean ± SD of six replicates. Different letters (a–e) denote statistically significant differences within each time according to Tukey’s test (*p* < 0.05).

Once the solvent was chosen, a time course of the cytotoxic effect of different concentrations of the SFE-NX extracts versus cell viability was determined (Figure 6). For this, HaCaT cells were seeded as described in Materials and Methods. After cellular attachment, the SFE-NX extracts were added to the wells to achieve final concentrations ranging from 0 to 200 µg extract mL^−1^ (equal to 46 µg of bioactive compounds mL^−1^) and incubated for 12, 24 and 48 h before the cell viability was measured. According to ANOVA analysis (Table S2), cell viability was significantly affected by the extract concentration, exposure time and the interaction between both factors. As shown in Figure 6, SFE-NX extracts appeared to be slightly toxic up to 50 µg extract mL^−1^, reducing cell viability by around 91% after 12 h of treatment. However, a significant decrease in cell viability was observed when HaCaT cells were exposed to extract concentrations greater than 50 µg extract mL^−1^ for 24 h (~83%), whereas SFE-NX extracts were cytotoxic at concentrations above 200 µg extract mL^−1^ at 48 h, when cell viability was reduced by up to 88%. The toxicity of the extracts reached a maximum at 24 h, showing the lowest IC_50_ value (256 µg extract mL^−1^) at 12 h (850 µg extract mL^−1^) and 48 h (1805 µg extract mL^−1^) after treatments.

**Figure 7:**
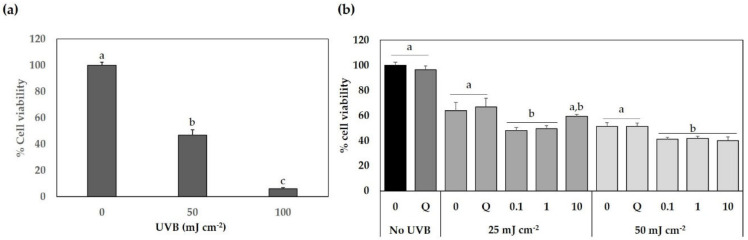
Effects of the optimized supercritical fluid (SFE) extracts derived from Naxos broccoli by-products (SFE-NX) on the viability of HaCaT keratinocytes exposed to ultraviolet B (UVB) light. In (**a**) HaCaT cells were irradiated with specific doses of UVB light (50 and 100 mJ cm^−2^) and cell viability was determined using an MTT assay after 24 h. In (**b**) HaCaT cells were pre-treated with different concentrations of SFE-NX extract (0.1, 1 and 10 µg extract mL^−1^), exposed to UVB light (25 and 50 mJ cm^−2^) and cell viability was measured after 24 h. Q: quercetin (30 µg mL^−1^, positive control). Cell viability is expressed as a percentage of the control. Different letters (a–c) denote statistically significant differences within UVB light doses according to Tukey’s test (*p* < 0.05).

After ascertaining the SFE-NX concentrations (≤10 µg extract mL^−1^) and exposure time (12 h) that did not significantly reduce cell viability, the protective effect of the SFE-NX extracts against UVB light was tested in HaCaT cells. First, HaCaT cells were exposed to specific doses of UVB light (0, 50 and 100 mJ cm^−2^) and incubated for 24 h. As shown in Figure 7a the exposure to UVB light resulted in a significant reduction of HaCaT cell viability in a dose-dependent manner. Thus, since the UVB light doses reduced cell viability by more than 50%, 25 and 50 mJ cm^−2^ were selected for subsequent experiments. The HaCaT cells were pretreated with different concentrations of the SFE-NX extracts, ranging from 0 to 10 µg extract mL^−1^, for 12 h and exposed to UVB light irradiation (0, 25 and 50 mJ cm^−2^). Cell viability was determined 24 h later. According to ANOVA analysis (Table S2), HaCaT cell viability was significantly affected by the UVB light irradiation, SFE-NX extract concentration, and the interaction between both factors. The effects of the SFE-NX extracts on UVB light cytotoxicity are shown in Figure 7b. As can be observed, HaCaT cell viability decreased significantly after UVB light irradiation with respect to non-irradiated cells. At low doses (25 mJ cm^−2^), the application of SFE-NX extracts increased the viability of the irradiated cells in a dose-dependent manner and no significant differences were found with respect to the control and quercetin-treated cells (*p* > 0.05) at 10 µg mL^−1^. These results suggest that a high concentration of SFE-NX extract (10 µg mL^−1^) could have a small cytoprotective effect on HaCaT cells exposed to UVB light. On the contrary, after irradiation with high doses of UVB light (50 mJ cm^−2^), no positive effects on cell viability were detected, even in the presence of a high concentration of SFE-NX.

One of the main skin-aging agents is UV, particularly UVB light, which is the most damaging component of solar radiation reaching the surface of the Earth. UVB light causes a broad range of skin lesions, including skin inflammation, photo-aging, and photo-carcinogenesis [37]. Several studies have demonstrated the protective effect of plant extracts against UVB light irradiation. For example, Shibata et al. [38] showed that pre-treatment of HaCaT cells with sulforaphane (1–25 µM), an isothiocyanate found in *Brassica* vegetables, recovered the loss of viability induced by UVB light (50 mJ cm^−2^) and suppressed the secretion of IL-6, an inflammatory marker whose production increases after UVB light exposure. Studies carried out by Park et al. [39] demonstrated that plant extracts derived from *Gardenia jasminoides* (100 µg extract mL^−1^) had a protective effect against UVB light-induced cell death in HaCaT cells at 10 mJ cm^−2^, while extracts from *Phellodendro amurense* and *Rheum rhabarbarum* had a lesser or no cytoprotective effect, respectively, in these conditions. Likewise, Vostálová et al. [40] evaluated the potential of a rosmarinic acid-enriched extract from *Prunella vulgaris* to suppress UVB light-induced alterations in human HaCaT keratinocytes. They found that pre-treatment with *P. vulgaris* extracts had a protective effect on HaCaT cells irradiated with UVB light. Similarly, Zaid et al. [41] showed that pre-treatment of HaCaT cells with pomegranate fruit extracts enriched in polyphenols (10–40 µg mL^−1^) inhibited the decrease in cell viability mediated by UVB light (15–30 mJ cm^−2^) by increasing the intracellular glutathione content and decreasing both lipid peroxidation and markers of photo-aging, thereby protecting HaCaT cells against UVB-light-induced oxidative stress. Calò and Marabini [42] also demonstrated that extracts enriched in polyphenols and anthocyanins from *Vaccinium myrtillus* were able to reduce UVB light-induced cytotoxicity and genotoxicity at lower doses of irradiation (32–64 mJ cm^−2^).

## 4. Conclusions

The last few years have seen several improvements in the strategies of extraction and analysis of bioactive compounds from different plant materials. The application of faster and more efficient extraction techniques, such as SFE, has considerably enhanced the yield of these compounds while significantly reducing organic solvent consumption. The overall quality of the extracts has also been improved, and a reduction in manipulation errors has led to better extraction reproducibility and repeatability. For all these reasons, SFE technology represents an effective alternative to conventional extraction techniques to obtain bioactive components from plant by-products. In this work, an experimental design based on RSM was developed to optimize and validate an SFE procedure, which was tested with broccoli by-products. The results show that the extracts obtained from Naxos broccoli by-products using SFE technology were far richer in total bioactive compounds than the conventional extracts, which correlated with their higher antioxidant activity. The protective effect of the SFE-NX extracts against UVB light was established in HaCaT cells, using concentrations (≤10 µg extract mL^−1^) and an exposure time (12 h) that did not significantly reduce cell viability. Their antioxidant activity and attenuation of the negative effect of UVB light on HaCaT cells indicates SFE-NX extracts have potential application as a cosmeceutical ingredient.

## Figures and Tables

**Table 1:** Values of independent factors used for the central composite design and response values. Data are given as the mean ± SD of two replicates.

Condition	Temperature (°C)	Pressure (bar)	% EtOH	Flow Rate (g min^−1^)	Time (min)	Response (mg β-Carotene g^−1^ Extract)
*SFE-1*	40	450	30	28	80	45.17 ± 1.23
*SFE-2*	60	150	18.5	30	60	48.53 ± 0.55
*SFE-3*	40	450	7	32	80	96.34 ± 3.26
*SFE-4*	60	300	18.5	30	40	41.22 ± 3.32
*SFE-5*	60	300	18.5	30	60	50.04 ± 3.45
*SFE-6*	60	300	18.5	30	80	75.08 ± 4.17
*SFE-7*	60	300	7	30	60	34.94 ± 0.19
*SFE-8*	40	450	30	32	40	66.51 ± 3.18
*SFE-9*	80	150	7	32	80	57.67 ± 1.48
*SFE-10*	40	150	30	28	40	11.51 ± 1.10
*SFE-11*	80	450	30	28	40	40.72 ± 0.63
*SFE-12*	60	300	18.5	28	60	29.42 ± 2.08
*SFE-13*	40	450	7	28	40	28.76 ± 0.27
*SFE-14*	40	150	30	32	80	53.55 ± 2.44
*SFE-15*	80	150	30	32	40	50.16 ± 0.38
*SFE-16*	80	450	30	32	80	28.41 ± 0.99
*SFE-17*	40	150	7	32	40	25.83 ± 0.58
*SFE-18*	80	450	7	32	40	38.88 ± 1.64
*SFE-19*	60	300	30	30	60	45.19 ± 0.60
*SFE-20*	80	150	30	28	80	30.95 ± 0.08
*SFE-21*	80	450	7	28	80	24.81 ± 0.77
*SFE-22*	40	300	18.5	30	60	65.54 ± 1.31
*SFE-23*	40	150	7	28	80	49.52 ± 1.40
*SFE-24*	80	300	18.5	30	60	31.26 ± 2.00
*SFE-25*	60	300	18.5	32	60	30.52 ± 2.44
*SFE-26*	60	300	18.5	30	60	46.76 ± 1.56

**Table 2:** Analysis of variance (ANOVA) and fitting parameters of the proposed CCD 2^5^ model. * Significant coefficients (*p* < 0.05 or *p* < 0.10). SS = sum of squares; Df = degrees of freedom; MS = mean square; R^2^ = Quadratic correlation coefficient.

Source	Y= β-Carotene Content				
	SS	Df	MS	F-Ratio	*p*-Value
X_1_: Temperature	1064.38	1	1064.38	197.87	0.0452 *
X_2_: Pressure	490.67	1	490.67	91.22	0.066 4*
X_3_: Ethanol	20.80	1	20.80	3.87	0.2995
X_4_: Flow rate	1870.99	1	1870.99	347.82	0.0341 *
X_5_: Time	1346.36	1	1346.36	250.29	0.0402 *
X_3_ X_5_	1282.13	1	1282.13	238.35	0.0412 *
X_4_ X_4_	604.38	1	604.38	112.35	0.0479 *
Lack-of-fit	3262.24	4	191.90	35.67	0.1296
Pure error	5.38	1	5.38		
Total (corr.)	8172.58	25			
R^2^	0.935				

**Table 3:** Major bioactive compounds, total bioactive compounds and extraction yield in the conventional extracts (CE) and in the optimized SFE extracts from Parthenon (SFE-PT) and Naxos (SFE-NX) by-products. The data in parenthesis represent the proportion of each type of compound with respect to the total bioactive compounds, expressed as %. Values are given as the mean ± SD of three replicates.

	Phytosterols (mg g^−1^)	α-Tocopherol (mg g^−1^)	Chlorophylls (mg g^−1^)	β-Carotene (mg g^−1^)	Phenolic Compounds (mg GAE g^−1^)	Total Bioactive Compounds (mg g^−1^)	Extraction Yield (%)
CE	25.04 ± 0.70 (6.38)	9.45 ± 0.59 (2.41)	30.03 ± 0.89 (7.66)	253. 40 ± 7.30 (64.61)	74.31 ± 1.02 (18.94)	392.22 ± 10.51	2.57
SFE-PT	19.71 ± 2.39 (12.19)	5.69 ± 0.13 (3.52)	32.64 ± 1.72 (20.19)	84.11 ± 1.51 (52.03)	19.51± 0.60 (12.07)	161.66 ± 6.35	2.61
SFE-NX	12.68 ± 1.43 (5.34)	5.67 ± 0.19 (2.39)	40.89 ± 2.19 (17.22)	148.93 ± 8.56 (62.74)	29.21 ± 5.04 (12.31)	237.38 ± 17.42	2.73

## Supplementary Materials

The following are available online at https://www.mdpi.com/2076-3921/9/12/1195/s1, Table S1. Comparison between experimental results and predicted values for SFE. CV= Coefficient of variance. Table S2: *F*-values from two-way ANOVA significant at the 99.9% (***), 99% (**), or 95% (*) level of probability.
