# Supplementary figures and images for: Revalorization of Broccoli By-Products for Cosmetic Uses Using Supercritical Fluid Extraction
Source: Antioxidants (Basel). 2020 Nov 27;9(12):1195. doi: 10.3390/antiox9121195 (PMC7760773; doi:10.3390/antiox9121195)

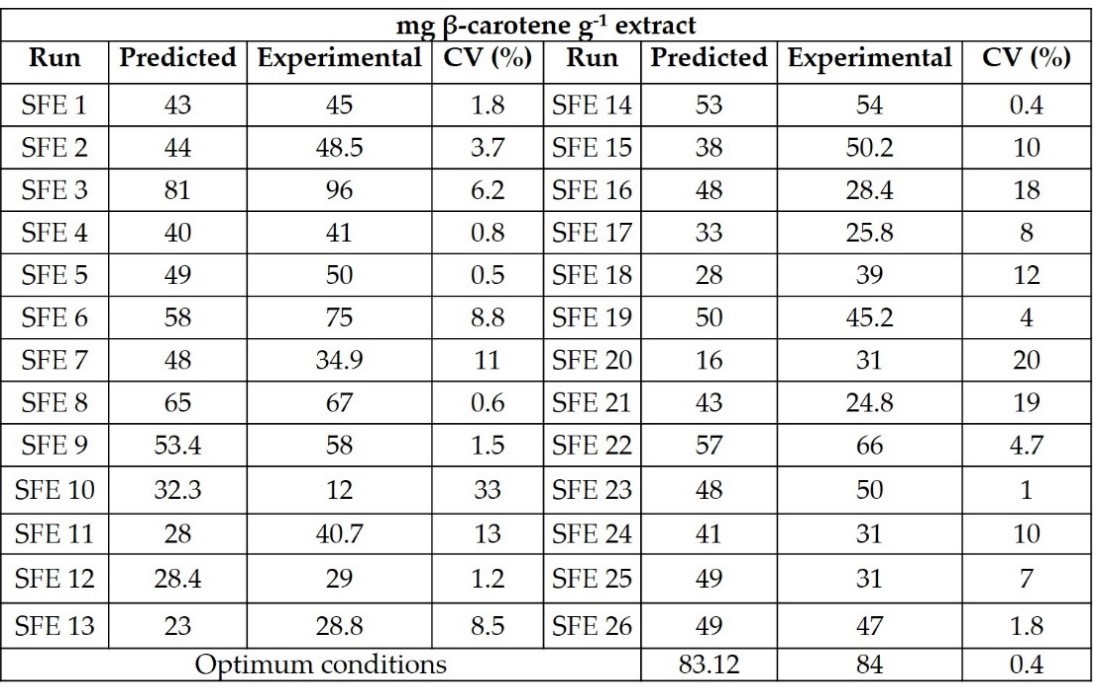

Supplement: Supplementary file 1 [file antioxidants-09-01195-s001.zip › Table S1.jpg]

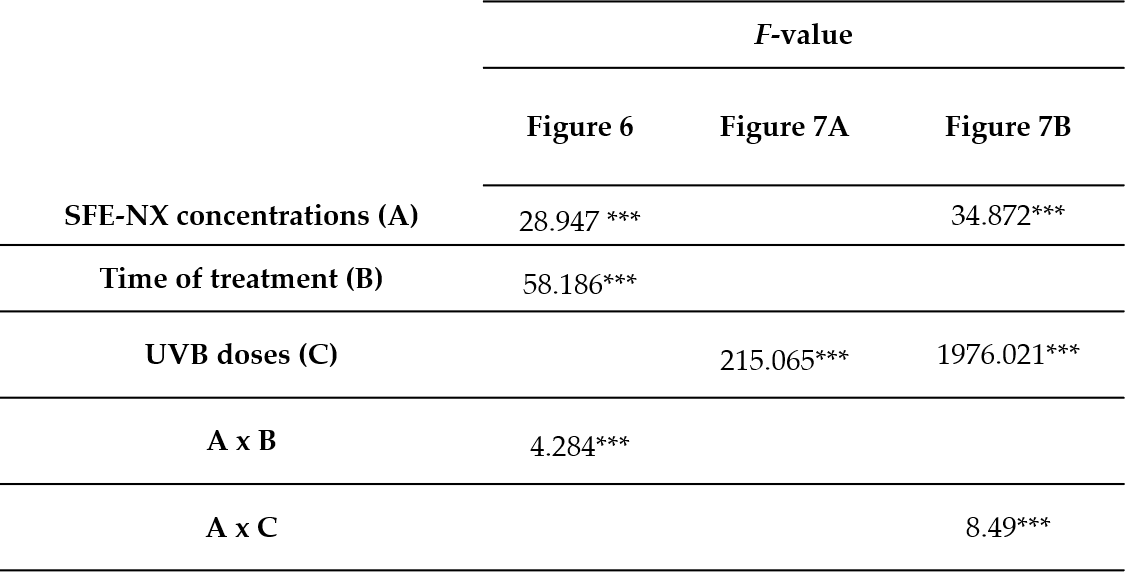

Supplement: Supplementary file 1 [file antioxidants-09-01195-s001.zip › Table S2.png]
